# The UAE healthy future study: a pilot for a prospective cohort study of 20,000 United Arab Emirates nationals

**DOI:** 10.1186/s12889-017-5012-2

**Published:** 2018-01-05

**Authors:** Abdishakur Abdulle, Abdullah Alnaeemi, Abdullah Aljunaibi, Abdulrahman Al Ali, Khaled Al Saedi, Eiman Al Zaabi, Naima Oumeziane, Marina Al Bastaki, Mohammed Al-Houqani, Fatma Al Maskari, Ayesha Al Dhaheri, Syed M. Shah, Tom Loney, Mohamed El-Sadig, Abderrahim Oulhaj, Leila Abdel Wareth, Wael Al Mahmeed, Habiba Alsafar, Benjamin Hirsch, Fatme Al Anouti, Jamila Yaaqoub, Claire K. Inman, Aisha Al Hamiz, Ayesha Al Hosani, Muna Haji, Teeb Alsharid, Thekra Al Zaabi, Fatima Al Maisary, Divya Galani, Tim Sprosen, Omar El Shahawy, Jiyoung Ahn, Tomas Kirchhoff, Ravichandran Ramasamy, Ann Marie Schmidt, Richard Hayes, Scott Sherman, Raghib Ali

**Affiliations:** 1 New York University Abu Dhabi, Abu Dhabi, United Arab Emirates; 2 Zayed Military Hospital, Abu Dhabi, United Arab Emirates; 3 Sheikh Khalifa Medical City, Abu Dhabi, United Arab Emirates; 4 UAE University, Al-Ain, United Arab Emirates; 5 Cleveland Clinic Abu Dhabi, Abu Dhabi, United Arab Emirates; 6 Khalifa University of Science Technology & Research, Abu Dhabi, United Arab Emirates; 7 Zayed University, Abu Dhabi, United Arab Emirates; 8 Abu Dhabi Police Medical Services, Abu Dhabi, United Arab Emirates; 9 University of Oxford, Oxford, UK; 10 New York University School of Medicine, New York, NY USA

**Keywords:** Adult, Chronic disease, Cohort studies, Pilot projects, Prospective studies, Public health, United Arab Emirates

## Abstract

**Background:**

The United Arab Emirates (UAE) is faced with a rapidly increasing burden of non-communicable diseases including obesity, diabetes, and cardiovascular disease. The UAE Healthy Future study is a prospective cohort designed to identify associations between risk factors and these diseases amongst Emiratis. The study will enroll 20,000 UAE nationals aged ≥18 years. Environmental and genetic risk factors will be characterized and participants will be followed for future disease events. As this was the first time a prospective cohort study was being planned in the UAE, a pilot study was conducted in 2015 with the primary aim of establishing the feasibility of conducting the study. Other objectives were to evaluate the implementation of the main study protocols, and to build adequate capacity to conduct advanced clinical laboratory analyses.

**Methods:**

Seven hundred sixty nine UAE nationals aged ≥18 years were invited to participate voluntarily in the pilot study. Participants signed an informed consent, completed a detailed questionnaire, provided random blood, urine, and mouthwash samples and were assessed for a series of clinical measures. All specimens were transported to the New York University Abu Dhabi laboratories where samples were processed and analyzed for routine chemistry and hematology. Plasma, serum, and a small whole blood sample for DNA extraction were aliquoted and stored at −80 °C for future analyses.

**Results:**

Overall, 517 Emirati men and women agreed to participate (68% response rate). Of the total participants, 495 (95.0%), 430 (82.2%), and 492 (94.4%), completed the questionnaire, physical measurements, and provided biological samples, respectively.

**Conclusions:**

The pilot study demonstrated the feasibility of recruitment and completion of the study protocols for the first large-scale cohort study designed to identify emerging risk factors for the major non-communicable diseases in the region.

**Electronic supplementary material:**

The online version of this article (10.1186/s12889-017-5012-2) contains supplementary material, which is available to authorized users.

## Background

The United Arab Emirates (UAE), a high-income developing country, has undergone a rapid epidemiological transition from a traditional semi-nomadic society to a modern affluent society with a lifestyle characterized by over-consumption of energy-dense foods and low physical activity [1–3]. As a result, the UAE is facing an increasing burden of non-communicable diseases (NCD) including obesity, diabetes, and cardiovascular disease (CVD).

More than a quarter (29%) of all deaths in the UAE are attributable to cardiovascular disease (CVD) [4], with a high prevalence of cardiometabolic risk factors [5]. Recent population-wide data showed that over 65% of adults are either overweight or obese, with 57% having central obesity [6]. Compared to the United States, one of the countries with the highest obesity burden, the rate of pre-diabetes or diabetes in the UAE, is far higher at 44% [1], according to the criteria from the American Diabetes Association (ADA).

This increasing burden of non-communicable diseases (NCDs) is largely attributable to complex co-determinants and risk factors including individual health habits (e.g., smoking, diet, and physical activity) [7], environmental factors [8], genetic susceptibility [9], human microbial milieu (microbiome) [10], and socio-economic conditions [11].

For example, one-quarter of men were reported to regularly smoke; mostly cigarettes and to a lesser extent Midwakh and water pipe (hookah/shisha) [12]. The use of tobacco is also increasing rapidly among adolescents [13]. In addition, poor nutritional intake and physical inactivity are common, particularly among females [2]. The consumption of protective dietary factors (e.g. fruits, vegetables, nuts, whole grains) is suboptimal, whereas consumption of harmful foods (e.g. processed meat, red meet, and trans fatty acids) are above recommended levels [5]. Despite the lack of population-wide studies using objective measures of free-living physical activity, low levels of physical activity, mostly due to cultural and climate restrictions, have been self-reported in adolescent females [14].

To date, no large prospective cohort studies have been carried out in the UAE, hence, the relative importance of both established and novel risk factors for NCDs is largely unexamined (A small sample of Emiratis was recruited into the PURE study [15]). Therefore, the UAE Healthy Future study (AEHFS), a prospective longitudinal cohort, is designed to recruit 20,000 healthy UAE nationals. For the sample size calculation, we focus on risk factors for Diabetes. The 20,000 figure is the minimum number that will enable us to quantify associations between exposures such as physical activity, BMI, Vitamin D levels and incident diabetes. As this is a prospective cohort study whose primary objective is to understand the association between risk factors and outcomes, a fully representative sample is not required. Thus we are recruiting a convenience sample - this is the same approach taken by many similar cohort studies including, for example, the UK biobank & Qatar biobank studies [17, 25].

Essentially, we conducted a pilot study to establish both the study feasibility and to ensure that all protocols and participant materials were appropriate for the local population.

The pilot study had five main objectives: 1) develop participant materials including the information leaflet, consent form, and questionnaires; 2) assess participation rates for various recruitment strategies; 3) evaluate the baseline assessment visit; 4) assess procedures for biological sample collection, transport and processing; and 5) assess participant acceptability of the individual study components. Here, we present our experience of conducting the pilot study and the results in relation to these five objectives.

### Subjects and methods

#### Subjects

The AEHFS is designed to collect detailed self-reported data in a questionnaire, take physical measurements, and collect biological specimens - blood, urine, and mouthwash samples from 20,000 UAE nationals aged ≥18 years. To test the feasibility of the study, we recruited 500 subjects. None of the participants received any incentives.

## Methods

### Development of participant materials

The first stage of the pilot study (September to December 2014) was a series of focus group discussions with potential participants to develop and optimize the participants’ information material and study protocols, the results of which has been published elsewhere [16].

After completion of the focus group discussions, we revised materials and carried out the actual pilot study as described here.

### Assessment of participation rates for various recruitment strategies

Making use of modifications suggested from the focus groups, the second stage of the pilot (actual recruitment of 500 participants into the cohort study) was carried out from January to April 2015 at two sites: Zayed Military Primary Health Care Clinic (ZMH PHCC) and the Abu Dhabi Blood Bank (ADBB), both of which are licensed for clinical research by the Health Authority of Abu Dhabi (HAAD). In each location, individuals who visited the clinic either for bi-annual medical screening (at the ZMH PHCC) or to donate blood (at ADBB) were invited to participate in the study. About 100 UAE nationals visit the ZMH PHCC daily whereas the ADBB receives about 20 UAE nationals daily on average.

All potential participants were given participant information leaflets in either Arabic or English to read and had the opportunity to ask questions prior to completion of the recruitment process. The age and gender of non-respondents, and their reasons for not participating was also recorded.

### Evaluation of baseline assessment visit

All participants were asked to complete a self-administered questionnaire on a touch screen tablet computer. Many of the questions were similar to the UK Biobank Study, a prospective cohort study of 500,000 adults in the UK [17]. The baseline questionnaire focused on known and potential risk factors for important public health outcomes including obesity, diabetes, and CVD [18]. These included questions on socio-demographic factors, lifestyle/habits, tobacco use, family history and early life exposures, general health, and psychological state (using PHQ-8, the Personal Health Questionnaire Depression Scale) [19, 20]. In the pilot study, we used the International Physical Activity Questionnaires (IPAQ) to measure physical activity.

Questions were selected based on their relevance, reliability, simplicity, and likelihood to show plausible associations with NCDs. Culturally or religiously sensitive questions such as questions on alcohol consumption – which is prohibited for Muslims in the UAE - were excluded.

The questionnaire was translated from English into Arabic and back-translated into English to check for linguistic validity. For the Personal Health Questionnaire Depression Scale (PHQ-8) and the STOP-BANG Questionnaire for Obstructive Sleep Apnea we used previously validated Arabic translations [21]. The time taken to complete the questionnaire and the response distributions for each question were examined. In a face-to-face interview, administered by a trained nurse, participants were asked about their medication history, past operations and other past medical history; and their fasting status. A summary of the questionnaire components is shown is Table 1. Dietary assessment will be conducted in the main study through the use of an online 24-h recall being developed for the use in the UAE. The full questionnaire is given in Additional file 1.

**Table 1:** Summary of baseline questionnaire components of the UAE healthy future pilot study

Measurement category	Instrument detail
Self-reported Questionnaires	
Sociodemographic	Employment status, Marital status, education, income, car ownership, and household size including workers.
Family history and early life exposures	Family history of major diseases, birth weight, breastfeeding, parental smoking, childhood body size, residence at birth
Psychosocial factors	Anxiety, depression, social support
Environmental factors	Current address, current (or last) occupation, housing, means of travel, shift work
Lifestyle	Smoking, physical activity, diet, sleep
Health status	Medical history, medications, disability, reproductive history (women)
Nurse interview questionnaire	Current and previous clinical history of the participants and and use of prescription medications

### Physical and clinical measurements

We measured sitting and standing height using a stadiometer (Seca, HamburgDeutschland) and neck, hip, and waist circumferences using a standard tape according to international guidelines [22]. Body mass and bio-impedance were measured and body mass index (BMI) was automatically calculated as body weight (kg)/ height square (meters) using the Tanita MC 780 (Tanita Inc., Tokyo, Japan).

Body weight was categorized based on BMI readings [underweight (< 18.5 kg/m^2^), normal range (18.5–24.9 kg/m^2^), overweight (25.0–29.9 kg/m^2^), obese class I (30.0–34.9 kg/m^2^), obese class II (35.0–39.9 kg/m^2^), obese class III (≥ 40.0 kg/m^2^)] according to world Health Organization categories. Pre-diabetes and diabetes were classified according to HbA1c levels: Normal: HBA1C < 5.7%; Pre-diabetes (5.7–6.4%); Diabetes HBA1c > 6.5%. Hypertension was classified as normal: SBP (90–119 mmHg) and DBP (60–79 mmHg), Prehypertension: SBP (120–139 mmHg) or DBP (80–89 mmHg), Stage 1 hypertension: SBP (140–159 mmHg) or DBP (90–99 mmHg), Stage 2 hypertension: SBP ≥ 160 or DBP ≥ 100 mmHg [23]. Hypercholesterolemia was defined by total cholesterol levels: desirable <200 mg/dl, borderline high 200–239 mg/dl, and high <240 mg/dl [24].

Hand-grip strength measurements were taken three times on participants’ right and left hand using a hydraulic hand dynamometer (Jamar Plus Digital Hand Dynamometer J00105, Jamar, USA) [22], and the highest reading was recorded for each hand-grip. Brachial blood pressure readings were taken twice on the upper left arm with appropriate cuff size with a two-minute interval between readings using a semi-automated sphygmomanometer (Omron M10-IT, Omron corporation, Kyoto, Japan). If the difference in blood pressure readings was ≥5 mmHg, then a third reading was obtained. A summary of the physical measurements components is shown is Table 2.

**Table 2:** Summary of Physical Measurements of the UAE Healthy Future pilot study

Measurement category	Instrument detail
Physical Measurements	
Anthropometrics	Standing and sitting height (Seca 202, Germany), body mass and bio impedance analysis (Tanita MC-780 MA body analyzer, Tanita Corporation, Tokyo, Japan), neck, waist, and hip circumference (Wessex non-stretchable sprung tape).
Hand grip strength	Right and left-hand grip strengths (Jamar hydraulic hand dynamometer, Patterson Medical, IL, USA).
Peripheral blood pressure and heart rate	Blood pressure measured twice (Omron M10-IT, Omron corporation, Kyoto, Japan)

### Assessment of procedures for biological sample collection, transport, and processing

To ensure that all sample handling and analyses could be completed at NYUAD, laboratories for clinical chemistry, hematology, and immunology were established prior to the actual recruitment process.

At the assessment centre, a unique barcode was generated for each individual. Samples, along with participants’ signed consent forms were labelled accordingly.

Participants provided several non-fasting specimens including blood (8 ml SST tube, and 8 ml Plasma EDTA tube), mouthwash in10 ml of physiological normal saline (0.9%), and the urine sample (approximately 10 ml) in a sterile screw-capped urine container. SST tubes were centrifuged (3500 rpm, 4 °C, for 15 min), but not separated, within 30 min from the time of collection. All samples were refrigerated (4 °C - 8 °C) and subsequently transported to NYUAD laboratories in a temperature-controlled cooler with adequate spill control contingency plans. On arrival, SST samples were aliquoted in 1.5 ml Eppendorf tubes. A proportion of the fresh serum was analyzed for routine chemistry using Beckman DXC 600 (Beckman, USA). Regarding the EDTA tubes, a small whole blood sample was aliquoted and stored at −80 °C for future DNA extraction prior to sample separation. Remaining EDTA sample, was centrifuged (3500 rpm, 4 °C, for 15 min). Plasma ETDA was aliquoted in 1.5 ml Eppendorf tubes and stored at −80 °C. A complete list of the biochemical markers being analyzed is summarized in Table 3.

**Table 3:** List of routine and advanced biochemical markers measured

Variable Category	Analyte
Bone markers	Calcium
	Phosphorus
	Uric acid
	Vitamin D
Diabetes	Glucose
	Glycated hemoglobin A1c % (HBA1c %) %
	Serum receptor for advanced glycation end-products (RAGE) - endogenous secretory (es)RAGE and soluble (s)RAGE
	Carboxymethil-lysine
	Homeostatic model assessment - insulin resistance (HOMA-IR) test on fasting participants
Electrolytes and renal function tests	Chloride
Electrolytes and renal function tests	Serum creatinine
	Potassium
	Sodium
	Urea nitrogen
Full blood count	Full blood count
Inflammation markers	C-Reactive protein
Lipid profile	Cholesterol
	High density lipoprotein
	Low density lipoprotein
	Triglycerides
	Apolipoprotein A
	Apolipoprotein B
Liver function tests	Albumin
	Alkaline phosphatase
	Alanine transaminase
	Aspartate transaminase
	Gamma glutamyl transferase
	Total bilirubin
	Total protein
Minerals	Iron
	Magnesium
Urine	Potassium
	Sodium
	Nitrogen
	Micro albumin
	Enzymatic Creatinine
	Cotinine

### Assessment of acceptability of various parts of the study

A brief post-visit questionnaire was sent to all participants by email or Short Message Service (SMS) to assess the acceptability of, and seek feedback on, the different components of the study.

### Analyses of data collected in the pilot study

Simple descriptive analyses of selected questions, physical measurements, and biomarkers collected in the pilot study were carried out to help inform the study protocol for the main study.

## Results

Overall, 517 participants were recruited over four months from January to April 2015. The response rate was 68% (517/769) and was similar in both recruitment sites. The primary reasons given for not taking part in the study were lack of time (60%), lack of interest (20%), not convinced e.g. benefits of study (10%), or no particular reasons (10%). There was no significant difference between the respondents and non-respondents in terms of age, gender, or occupation.

Table 4 shows the breakdown of the time taken to complete the different parts of the assessment visit. On average, the visit took between 40 to 60 min. Of the 517 participants, 495 (95%) filled out the questionnaire (although only 80% completed all sections), 430 (82%) had their physical measurements taken, and 492 (94%) provided at least one biological specimen -, 462 (89%) blood samples (plasma and serum), 389 (75%) urine sample, and 409 (79%) mouthwash sample. Many participants expressed the view that the questionnaire was too long.

**Table 4:** Time spent in the different recruitment stations

Visit Station	Assessments	Time taken (mins)
Reception & consent	• Welcome and given a printed copy of the Participant Information Leaflet (Arabic and English) • Consent taken and form signed	5–10
Questionnaire	• Self-administered touch-screen questionnaire via tablet	15–25
Interview & Physical Measurements	• Interviewer questionnaire • Blood pressure measurement • Standing & sitting height • Body mass • Waist & hip circumference • Neck circumference • Bio-impedance measure • Hand-grip strength	15
Specimen Collection	• Collection of blood samples (about 14 ml) • Urine sample (50 ml) • Oral wash (10 ml)	5–10
Total (min)		40–60 min

Samples were transported to NYUAD laboratories within 24 h of the collection with no major adverse temperature effects. All tests performed at NYUAD laboratory were validated on site for accuracy, precision, linearity using Clinical Laboratory Standards Institute guidelines (CLSI- EP15A2, EP6A, EP9). Reference ranges validation were also carried out on normal subjects using the transference method published by CLSI (CLSI C28-A3). Internal Quality control materials were analysed with every analytical run to control the analytical precision using two levels, while external laboratory comparisons with Cleveland Clinic Abu Dhabi (CCAD) Laboratory were used to control for bias and inaccuracy. CCAD uses External Proficiency Quality Control material from the College of American Pathologists (CAP) and is accredited by CAP.

**Fig. 1:**
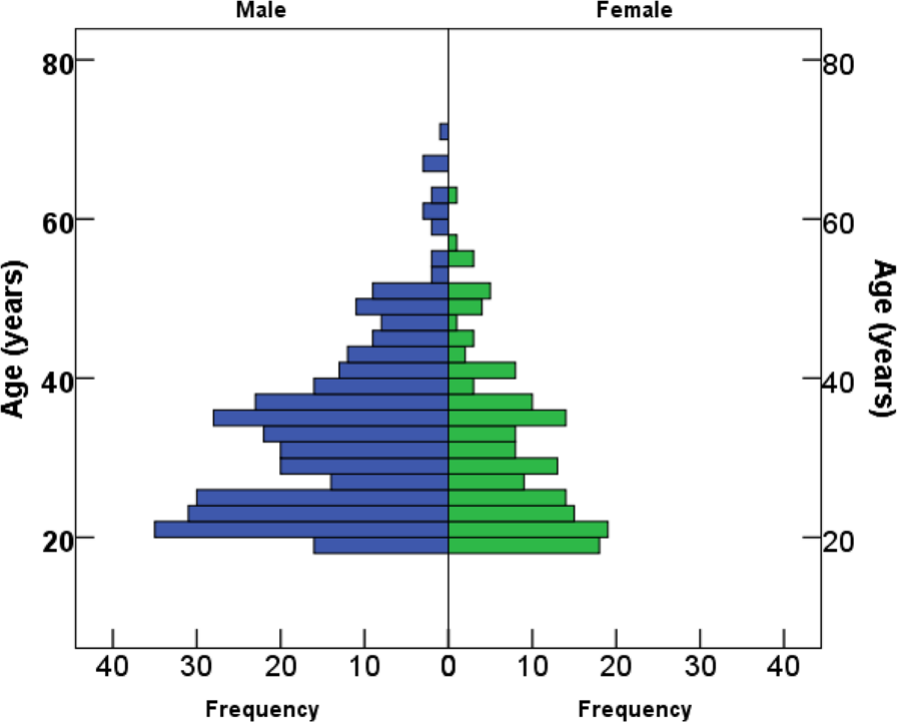
Age distribution of the pilot study participants (*n* = 495) among men (32.6 ± 10.6, *n* = 332) and women (30.1 ± 9.9, *n* = 159)

Of the 517 participants, 495 were included in the final analysis due to missing data. Age distribution ranged from 18 to 70 years (67% male) with the majority being below 40 years old among both males and females. The mean age ± SD of males (32.6 ± 10.7, *n* = 332) was significantly higher (*P* < 0.01) than that of females (30.0 ± 9.9, *n* = 159) as shown in Fig. 1.

Table 5 shows selected baseline socio-demographic characteristics among the participants of the pilot study, by sex.

**Table 5:** Selected baseline socio-demographic characteristics among the participants of the UAE Heath Future pilot study, by sex. (*N* = 491)

		Gender			
		Male		Female	
		*N*	%	*N*	%
Age Group	< 25	98	29.5	59	37.1
	25–34	104	31.3	55	34.6
	35–44	81	24.4	29	18.2
	45–54	37	11.1	12	7.5
	> 55	12	3.6	4	2.5
Education	Illiterate	7	2.2	1	0.7
	Primary school	41	13.1	5	3.3
	Secondary school	133	42.5	71	46.4
	University	105	33.5	68	44.4
	Postgraduate	13	4.2	4	2.6
	Prefer not to answer	10	3.2	2	1.3
	None of the above	4	1.3	2	1.3
Marital status	Single	107	34.2	81	52.9
	Married	196	62.6	57	37.3
	Divorced	10	3.2	12	7.8
	Widowed	0	0.0	3	2.0
Employment status	Paid or self-employed	163	52.1	62	40.5
	Retired	12	3.8	1	0.7
	Looking after home or family	41	13.1	23	15.0
	Unable sickness or disability	0	0.0	0	0.0
	Unemployed	10	3.2	17	11.1
	Unpaid or voluntary	3	1.0	0	0.0
	Student	22	7.0	22	14.4
	Prefer not to answer	33	10.5	16	10.5
	None of the above	29	9.3	12	7.8

Table 6 shows the crude (not age-adjusted) prevalence of self-reported chronic diseases among the participants stratified by sex.

**Table 6:** Crude (not age-adjusted) prevalence of self-reported chronic diseases among the participants of the UAE Health Future pilot study, by sex

	Male (*N* = 267)		Female (*N* = 140)	
	N	%	N	%
Age (mean ± SD)	33 (11)		30 (10)	
Diabetes	20	8	5	4
Hypertension	28	11	16	11
High Cholesterol	49	18	18	13
Current smoking				
Cigarette	51	18	1	1
Midwakh (local pipe)	52	18	1	1
Sheesha (water pipe)	41	14	2	1

**Fig. 2:**
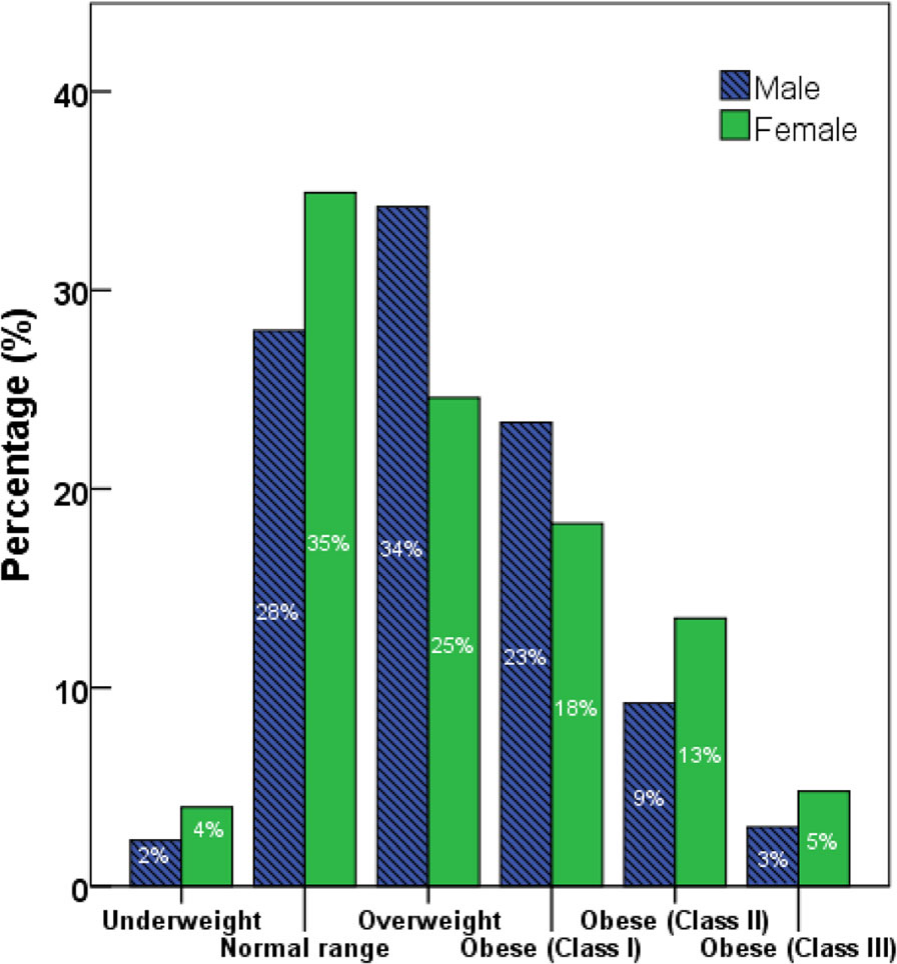
Body size categories based on body mass index cut-off points from the World Health Organization, by sex

**Fig. 3:**
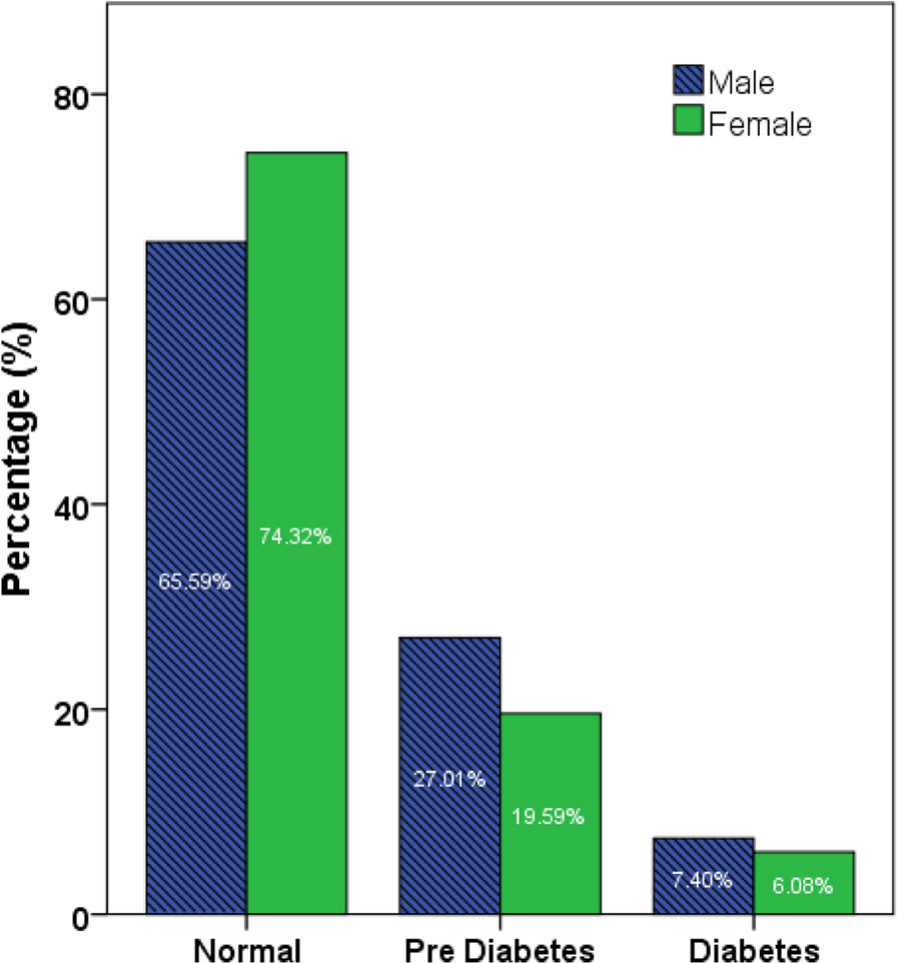
Prevalence of pre-diabetes (HBA1c 5.7 to 6.4%) and diabetes (>6.5%) according to HbA1c levels, by sex

**Fig. 4:**
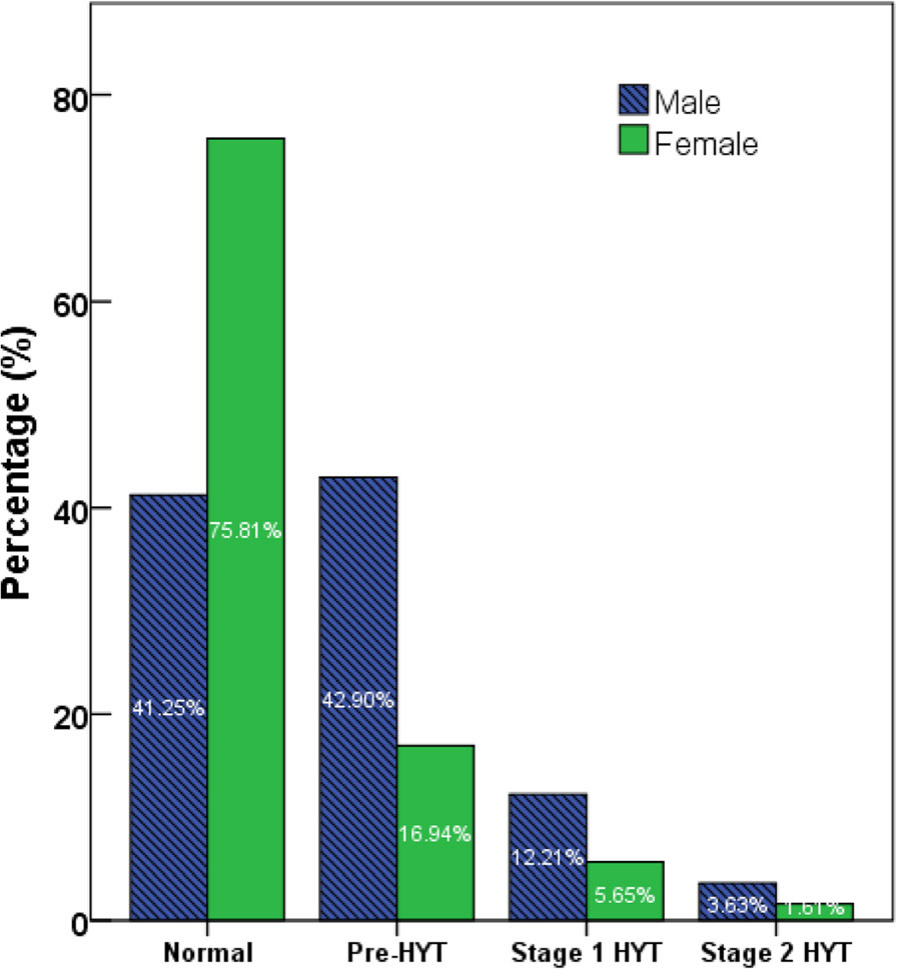
Prevalence of pre-hypertension and hypertension among the participants of the UAE Heath Future pilot study, by sex

**Fig. 5:**
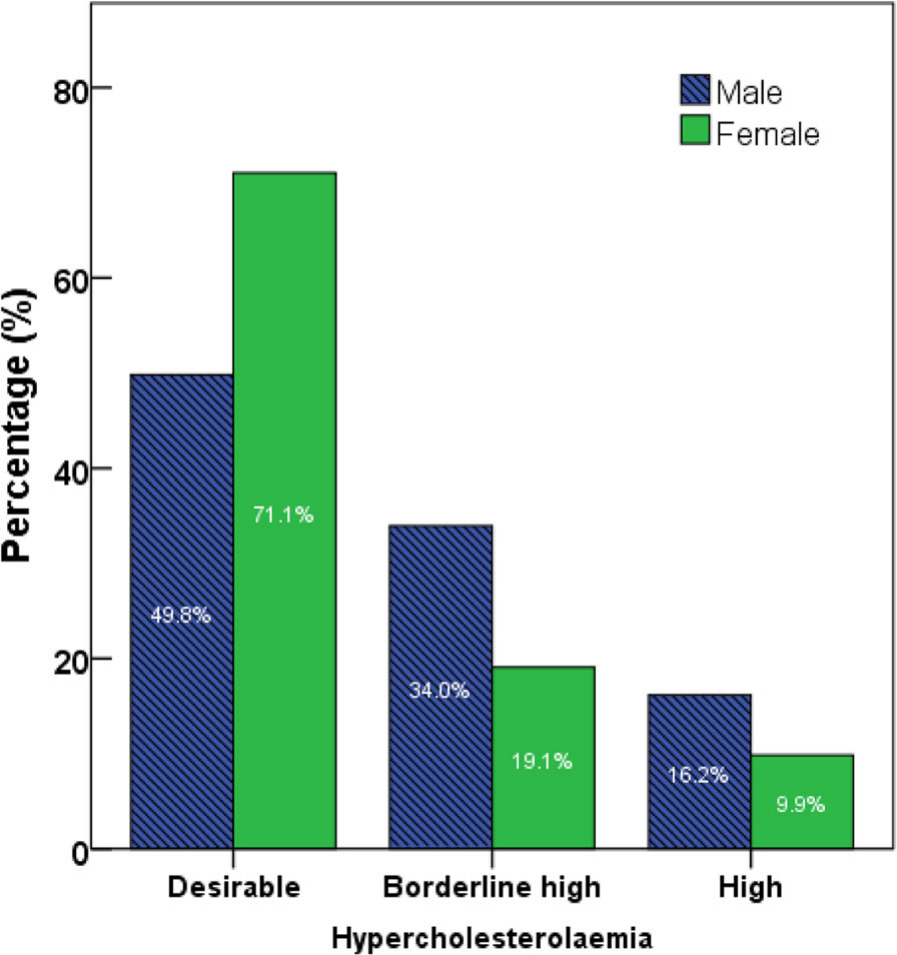
Prevalence of hypercholesterolemia among the participants of the UAE Heath Future pilot study, by sex

Figures 2, 3, 4, 5 show the prevalence of overweight and obesity, pre-diabetes and diabetes, hypertension, and hypercholesterolemia.

Table 7 shows the responses to the follow-up questionnaire. Of 330 questionnaires sent by email or SMS, 34 were completed (~10% response rate). Approximately 50% of the subjects reported that they fully read and understood the information leaflet. Half of the respondents thought that the length of the visit was adequate. However, 50% thought that the questionnaire time was too long. The majority of participants indicated that helping to improve the health of future Emirati generations, having a health check, or supporting medical research as main reasons for participation. Encouragingly, the overwhelming majority (80%) agreed that they would recommend a family member or friend to participate in the study.

**Table 7:** Responses to selected post recruitment follow-up questionnaire

Question	Response
Did you read the Participant Information Leaflet?	50% in detail 40% quick look 10% no
Was the length of the visit?	50% Just right 50% too long
Was the amount of information you were asked during the visit?	10% too short 40% Just right 50% too long
What were your main reasons for taking part in the UAEUHFS?	To help to improve the health of future generations. To have a health check. Support medical research.
How would you answer if a close friend or family member were to ask you “should I participate in the UAEUHFS?”	80% definitely take part 20% not sure

## Discussion

The AEHFS initiative provides for the first time a unique opportunity to identify causes and specific risk factors for NCDs among the Emirati population. The current pilot study was conducted to assess the feasibility of recruiting 20,000 UAE nationals by addressing five specific objectives including developing participants’ materials, assessing participation rates for various recruitment strategies, evaluating baseline visits, assessing procedures for biological sample handling and analyses of collected data, and assessing the acceptability of the study.

Our study results show that a high proportion of UAE nationals (68% response rate) are willing to participate in research aimed at improving the health of future generations. This high response rate establishes the feasibility of recruiting 20,000 participants for the main study. Furthermore, the highest proportion (60%) among subjects who refused to participate did so due to time constraints only, indicative of the general acceptability of the study protocols. Considering the time taken to complete the recruitment process, a research team of one nurse and one research assistant would be able to recruit about 10 individuals per day. Thus, we think that five recruitment clinics would suffice to recruit the target number of participants within two years. Based on the experience from the pilot study, a custom-made laboratory information management system (LIMS) was developed to monitor sample collection, transport, storage, and biochemical analysis.

The participants of our pilot study were relatively younger than in many other longitudinal studies internationally, and even within the region [25]. This young age cohort is useful mainly because of the incidence of our primary outcome of interest, diabetes, increases rapidly from the age of 40 years. Further, a younger cohort will allow us to understand the importance of risk factors operating from early adulthood. Our current results show significantly high levels of self-reported diabetes, hypertension, and hypercholesterolemia. As expected and previously reported [26], objective measures of the prevalence of these diseases were even higher particularly among males.

The percentage of participants who responded to our follow-up questionnaire (sent by SMS or email) was relatively low at 10% and may not be reflective of the whole sample. Overall, half of the respondents understood the information leaflet, perhaps as a reflection of the high literacy rate (90%) among participants. While patriotism was the single most important promoter to participation, the length of the questionnaire was an important obstacle, − thus, the length of the questionnaire will be reduced to improve completion rates.

## Conclusion

The pilot study successfully demonstrated the feasibility of the study and recruitment for the main study that has now started and is due to be completed in 2018. As the first prospective cohort study in Abu Dhabi, the AEHFS will provide unique and substantive evidence for both lifestyle and genetic determinants of common diseases in the Emirati population. The study will be a resource for researchers throughout the UAE, and the wider region, with data accessible for all research that will be of benefit to the local population.

## Additional file

Additional file 1: Participant Completed Questionnaire. (DOCX 82 kb)

## Abbreviations

ADBB: Abu Dhabi Blood Bank
AEHFS: UAE Healthy Future study
BMI: Body mass index
HAAD: Health Authority of Abu Dhabi
IPAQ: International Physical Activity Questionnaires
LIMS: Laboratory information management system
NCD: None communicable diseases
NYUAD: New York University Abu Dhabi
PHQ-8: Personal Health Questionnaire Depression Scale
SKMC: Sheikh Khalifa Medical City
STOP-BANG: Questionnaire for Obstructive Sleep Apnea
ZMH PHCC: Zayed Military Primary Health Care Clinic
ZMH: Zayed Military Hospital (ZMH)
