# Supplementary material for: The UAE healthy future study: a pilot for a prospective cohort study of 20,000 United Arab Emirates nationals
Source: BMC Public Health. 2018 Jan 5;18:101. doi: 10.1186/s12889-017-5012-2 (PMC5755402; doi:10.1186/s12889-017-5012-2)
Supplement: Additional file 1: — Participant Completed Questionnaire. (DOCX 82 kb) [file 12889_2017_5012_MOESM1_ESM.docx]

**Participant Completed Questionnaire**

| **Q. No** | **Stem** | **Responses / Categorical Label** |
| --- | --- | --- |
| **P1** | First Name  Family Name | Selection   - Free text |
| **P2** | Address/ PO Box | Selection   - Free text |
| **P3** | Date of birth: | Select from drop down menus |
| **P4** | Mobile number | Selection   - Free text |
| **P5** | Email address | Selection   - Free text |

| **Q. No** | **Stem** | **Responses / Categorical Label** |
| --- | --- | --- |
| **D1** | Gender | 01 Male  02 Female |
| **D2** | Marital Status | 01 Single 02 Married 03 Divorced/ separated 04 Widow/Widower |
| **D3A** | Emirate / city of residence | 01 Abu Dhabi  02 Al Ain 03 Dubai 04 Sharjah 05 Ajman 06 Um Al Quwain 07 Ras Al Khaima 08 Fujairah |
| **D3B** | **Area of residence** | Select one from **List of areas**  **NN None of above** UN Do not know DA Prefer not to answer |
| **D4A** | **How many houses do you own? (include farm house, Island house, beach house, house in different Emirate, house in different country)** | Enter number  OR  UN Do not know  OR  DA Prefer not to answer |
| **D5** | **Do you or your family own or rent the primary housing that you live in?** | Select one from   - 01 Own outright (by you or someone in your household) - 02 Own with a mortgage - 03 Rent - 04 Live in accomadation rent free / government granted housing - NN None of the above - DA Prefer not to answer |
| **D7** | **Including yourself, how many people are living together in your primary household? (Include those who usually live in the house such as students living away from home during term) Do not include people working in your household** | Enter number  OR  UN Do not know  OR  DA Prefer not to answer |
| D7A | **How are the other people who live with you related to you? (You can select more than one answer)** | Select from   - 01 Husband or wife - 02 Son and/or daughter (include step-children) - 03 Brother and/or sister - 04 Mother and/or father - 05 Grandparent - 06 Grandchild - 07 Other related - 08 Other unrelated - DA Prefer not to answer |
| D7B | **How many people are working in your primary household? (Please include drivers, housemaids, cooks, farmers, etc.)** | Enter number  OR  UN Do not know  OR  DA Prefer not to answer |
| D7C | **How many rooms are there in your house (not including kitchens and bathrooms)?** | Enter number  OR  UN Do not know  OR  DA Prefer not to answer |
| **D8** | **How many cars or vans are owned, or available for use, by you or members of your household? (please include company vehicles if available for private use)** | Enter number  OR  UN Do not know  OR  DA Prefer not to answer |
| **D10** | **What is the total monthly income received by your HOUSEHOLD in AED?** | Select one from   - 01 Less than 20,000 - 02 20,000 to 39,999 - 03 40,000 to 59,999 - 04 60,000 to 79,999 - 05 80,000 to 99,000 - 06 100,000 to 119,000 - 07 Greater than 120,000 - UN Do not know - DA Prefer not to answer |
| **D10A** | **How many times a year do you travel overseas for a holiday?** | Enter number  OR  UN Do not know  OR  DA Prefer not to answer |
| **D9** | **Which of the following describes your current situation? (You can select more than one answer)** | Select from   - 01 In paid employment, - 02 Self-employed (e.g. consultant) - 03 Business man / business women - 04 Employer - 05 Retired - 06 House wife - 07 Unable to work because of sickness or disability - 08 Unemployed - 09 Doing unpaid or voluntary work - 10 Full or part-time student - NN None of the above - DA Prefer not to answer |
| D9A1 | **Which of the following describes your occupation?** | Select from:   - 01 Manager - 02 Professional - 03 Technician or Associate professional - 04 Clerical support worker - 05 Service or sales worker - 06 Agricultural, forestry or fishery worker - 07 Craft or related trades - 08 Plant or machine operator - 09 Elementary occupation - NN None of the above - DA Prefer not to answer |
| D9A | **In a typical WEEK, how many hours do you spend at work? (Do not include hours travelling to and from work)** | Enter number  OR  UN Do not know  OR  DA Prefer not to answer |
| D9E | **What level of activity is involved in your main occupation?** | Select one from   - 01 Sitting most of the time - 02 Standing most of the time - 03 Walking most of the time - 04 Sitting, standing, and walking in equal amounts - 05 Other work with moderate physical activity (includes moving or lifting objects of moderate weight) - 06 Physically heavy work (includes moving or lifting heavy objects or activities) - UN Do not know - DA Prefer not to answer |
| D9F | **Does your work involve shift work?** | Select one from   - 01 Never/rarely - 02 Sometimes - 03 Usually - 04 Always - UN Do not know - DA Prefer not to answer |
| D9DA | **Does your job involve working during the night?** | Select one from   - 01 Never/rarely - 02 Sometimes - 03 Usually - 04 Always - UN Do not know - DA Prefer not to answer |
| **D10** | **For how many years have you not been working?** | - One or less - More than one year ago - Never worked before - Prefer not to answer |
| **D11** | **What is the highest level of education that you have completed?** | Select from   - 01 Did not attend or complete primary school - 02 Primary school - 03 Middle school - 03 Secondary school - 04 University - 05 Postgraduate degree (e.g Masters or PhD) - NN None of the above - DA Prefer not to answer |
| **D12** | **How old were when you/your family first owned your own house?** | Enter number  OR  Select one from   - 01From birth. - 02 We live in rented accommodation - UN Do not know - DA Prefer not to answer |
| **D12A** | **How old were you when your house first had an indoor toilet?** | Enter number  OR  Select one from   - 01 From birth. - 02 We don’t have an indoor toilet - UN Do not know - DA Prefer not to answer |
| **D12B** | **How old were you when your house first had Air Conditioning?** | Enter number  OR  Select one from   - 01 From birth. - 02 We don’t have AC - UN Do not know - DA Prefer not to answer |
| **D12C** | **How old were you when your household first had a car?** | Enter number  OR  Select one from   - 01 From birth. - 02 We don’t have a car - UN Do not know - DA Prefer not to answer |
| **D12D** | **How old were you when your household first had a housemaid /cook / driver/ gardener?** | Enter number  OR  Select one from   - 01 From birth. - 02 We don’t have a housemaid/ cook/ driver / gardener - UN Do not know - DA Prefer not to answer |
| **Q. No** | **Stem** | **Responses** |
| **D13** | **Where did you and your family live at around the time:** |  |
| **D13A** | **Of your birth** | Select one from   - 01 City - 02 Village - 03 Desert (bedouin) - 04 Island - 05 Other - UN Do not know - DA Prefer not to answer |
| **D13C** | **When you were 18 years old** | Select one from   - 01 City - 02 Village - 03 Desert (bedouin) - 04 Island - 05 Other - UN Do not know - DA Prefer not to answer |
|  | **Now** | Select one from   - 01 City - 02 Village - 03 Desert (bedouin) - 04 Island - 05 Other - UN Do not know   DA Prefer not to answer |
| **D14** | **In what type of housing did you live at around the time:** |  |
| **D14A** | **Of your birth** | Select one from   - 01 Villa - 02 Apartment - 03 Shabiyah - 04 Other - UN Do not know - DA Prefer not to answer |
| **D14C** | **When you were 18 years old** | Select one from   - 01 Villa - 02 Apartment - 03 Shabiyah - 04 Other - UN Do not know - DA Prefer not to answer |
|  | **Now** | Select one from   - 01 Villa - 02 Apartment - 03 Shabiyah - 04 Other - UN Do not know   DA Prefer not to answer |
| **D15** | **In a typical week, on how many days is incense burned in your home?** | - Never - One day per week or less - Frequently (2 -5 days per week) - Almost every day (6-7 days per week) - UN Do not know - DA Prefer not to answer |

**GPAQ**

| **Physical Activity** | | | | | | | | | | | |  |  |  |  |
| --- | --- | --- | --- | --- | --- | --- | --- | --- | --- | --- | --- | --- | --- | --- | --- |
| Next I am going to ask you about the time you spend doing different types of physical activity in a typical week. Please answer these questions even if you do not consider yourself to be a physically active person.  Think first about the time you spend doing work. Think of work as the things that you have to do such as paid or unpaid work, study/training, household chores, harvesting food/crops, fishing or hunting for food, seeking employment. *[Insert other examples if needed].* In answering the following questions 'vigorous-intensity activities' are activities that require hard physical effort and cause large increases in breathing or heart rate, 'moderate-intensity activities' are activities that require moderate physical effort and cause small increases in breathing or heart rate. | | | | | | | | | | | |  |  |  |  |
| **Questions** | | | | | | | **Response** | | | **Code** | |  |  |  |  |
| **Activity at work** | | | | | | | | | | | | | |  |  |
| 1 | | | Does your work involve vigorous-intensity activity that causes large increases in breathing or heart rate like *[carrying or lifting* *heavy loads, digging or construction work*] for at least 10 minutes continuously?  *[INSERT EXAMPLES] (USE SHOWCARD)* | | | Yes | | 1 | | | P1 | | |  |  |
|  |  |  |  |  |  | No | | 2  *If No, go to P 4* | | |  |  |  |  |  |
| 2 | | | In a typical week, on how many days do you do vigorous-intensity activities as part of your work? | | | Number of days | | └─┘ | | | P2 | | |  |  |
| 3 | | | How much time do you spend doing vigorous-intensity activities at work on a typical day? | | | Hours : minutes | | └─┴─┘: └─┴─┘  hrs mins | | | P3  (a-b) | | |  |  |
| 4 | | | Does your work involve moderate-intensity activity that causes small increases in breathing or heart rate such as brisk walking *[or carrying light loads*] for at least 10 minutes continuously?  *[INSERT EXAMPLES] (USE SHOWCARD)* | | | Yes | | | 1 | | | P4 | | |  |
|  |  |  |  |  |  | No | | | 2 *If No, go to P 7* | | |  |  |  |  |
| 5 | | | In a typical week, on how many days do you do moderate-intensity activities as part of your work? | | | Number of days | | | └─┘ | | | P5 | | |  |
| 6 | | | How much time do you spend doing moderate-intensity activities at work on a typical day? | | | Hours : minutes | | | └─┴─┘: └─┴─┘  hrs mins | | | P6  (a-b) | | |  |
| **Travel to and from places** | | | | | | | | | | | | | | | |
| The next questions exclude the physical activities at work that you have already mentioned.  Now I would like to ask you about the usual way you travel to and from places. For example to work, for shopping, to market, to place of worship. [insert other examples if needed] | | | | | | | | | | | | | | | |
| 7 | | | | Do you walk or use a bicycle (*pedal cycle*) for at least 10 minutes continuously to get to and from places? | | Yes | | | | | 1 | P7 | | |  |
|  |  |  |  |  |  | No | | | | | 2  *If No, go to P 10* |  |  |  |  |
| 8 | | | | In a typical week, on how many days do you walk or bicycle for at least 10 minutes continuously to get to and from places? | | Number of days | | | | | └─┘ | P8 | | |  |
| 9 | | | | How much time do you spend walking or bicycling for travel on a typical day? | | Hours : minutes | | | | | └─┴─┘: └─┴─┘  hrs mins | P9  (a-b) | | |  |
| **Recreational activities** | | | | | | | | | | | | | | |  |
| The next questions exclude the work and transport activities that you have already mentioned.  Now I would like to ask you about sports, fitness and recreational activities (leisure), [insert relevant terms]. | | | | | | | | | | | | | | |  |
| 10 | | | | Do you do any vigorous-intensity sports, fitness or recreational (*leisure*) activities that cause large increases in breathing or heart rate like [*running or football,]* for at least 10 minutes continuously?  *[INSERT EXAMPLES] (USE SHOWCARD)* | | | Yes | | | | 1 | | P10 | | |
|  |  |  |  |  |  |  | No | | | | 2  *If No, go to P 13* | |  |  |  |
| 11 | | | | In a typical week, on how many days do you do vigorous-intensity sports, fitness or recreational (*leisure*) activities? | | | Number of days | | | | └─┘ | | P11 | | |
| 12 | | | | How much time do you spend doing vigorous-intensity sports, fitness or recreational activities on a typical day? | | | Hours : minutes | | | | └─┴─┘: └─┴─┘  hrs mins | | P12  (a-b) | | |

*Continued on next page*

**GPAQ,** Continued

| **Physical Activity (recreational activities)** contd. | | | | | | |
| --- | --- | --- | --- | --- | --- | --- |
| **Questions** | | **Response** | | **Code** | | |
| 13 | Do you do any moderate-intensity sports, fitness or recreational *(leisure*) activities that causes a small increase in breathing or heart rate such as brisk walking*,*(*cycling, swimming, volleyball*)for at least 10 minutes continuously?  *[INSERT EXAMPLES] (USE SHOWCARD)* | Yes | 1 | | P13 | |
|  |  | No | 2  *If No, go to P16* | |  |  |
| 14 | In a typical week, on how many days do you do moderate-intensity sports, fitness or recreational (*leisure*) activities? | Number of days | └─┘ | | P14 | |
| 15 | How much time do you spend doing moderate-intensity sports, fitness or recreational (*leisure*) activities on a typical day? | Hours : minutes | └─┴─┘: └─┴─┘  hrs mins | | P15  (a-b) | |
| **Sedentary behaviour** | | | | | | |
| The following question is about sitting or reclining at work, at home, getting to and from places, or with friends including time spent [sitting at a desk, sitting with friends, travelling in car, bus, train, reading, playing cards or watching television], but do not include time spent sleeping.  *[INSERT EXAMPLES] (USE SHOWCARD)* | | | | | | |
| 16 | How much time do you usually spend sitting or reclining on a typical day? | Hours : minutes | └─┴─┘: └─┴─┘  hrs min s | | P16  (a-b) |  |

Activities

| **WP11** | **How often do you visit friends or family or have them visit you?** | Select one from   - 01 Almost daily - 02 2-4 times a week - 03 About once a week - 04 About once a month - 05 Once every few months - 06 Never or almost never - 07 No friends/family outside household - UN Do not know - DA Prefer not to answer |
| --- | --- | --- |
| **WP12** | **Which of the following do you attend once a week or more often? (You can select more than one)** | Select from   - 01 Sports club or gym - 02 Mosque - 03 Adult education class - 04 Family development foundation - 05 Other group activity - NN None of the above - DA Prefer not to answer |
| WP12A | **In a typical DAY in summer, how many hours do you spend outdoors and are exposed to the sun?** | Enter number  OR  FR Less than an hour a day  OR  UN Do not know  OR  DA Prefer not to answer |
| WP12B | **In a typical DAY in winter, how many hours do you spend outdoors and are exposed to the sun?** | Enter number  OR  FR Less than an hour a day  OR  UN Do not know  OR  DA Prefer not to answer |
| **WP5** | **In a typical DAY, how many hours do you spend watching TV or using a computer, tablet, smartphone(do not include using a computer at work; put 0 if you do not spend any time doing it)?** | Enter number  OR  FR Less than an hour a day  OR  UN Do not know  OR  DA Prefer not to answer |
| **WP7** | **In a typical DAY, how many hours do you spend driving?** | Enter number  OR  FR Less than an hour a day  OR  UN Do not know  OR  DA Prefer not to answer |

**Sleep**

| **SL1** | **About how many hours sleep do you get in every 24 hours? (please include naps)** | Enter number  OR  UN Do not know  OR  DA Prefer not to answer |
| --- | --- | --- |
| **SL1A** | **On an average day, how easy do you find getting up in the morning?** | Select one from   - 01 Not at all easy - 02 Not very easy - 03 Fairly easy - 04 Very easy - UN Do not know - DA Prefer not to answer |
| **SL1B** | **Do you have a nap during the day?** | Select one from   - 01 Never/Rarely - 02 Sometimes - 03 Usually - DA Prefer not to answer |
| **SL2** | **Do you have trouble falling asleep at night or do you wake up in the middle of the night?** | Select one from   - 01 Never/Rarely - 02 Sometimes - 03 Usually - DA Prefer not to answer |
| **SL4** | **Do you snore loudly (louder than talking or loud enough to be heard through closed doors)?** | Select one from   - 01Yes - 02 No - UN Do not know - DA Prefer not to answer |
| **SL5** | **Do you often feel tired, fatigued or sleepy during daytime?** | Select one from   - 01Yes - 02 No - UN Do not know - DA Prefer not to answer |
| **SL6** | **Has anyone observed you stopping breathing during your sleep?** | Select one from   - 01Yes - 02 No - UN Do not know - DA Prefer not to answer |

| Q. No | Stem | Responses |
| --- | --- | --- |
| INTRO 4 | Now, some questions about smoking and tobacco use..  Press ‘next’ to continue. | Only one selection  - Next |
| FR87225 | We will begin by asking you about your use of cigarettes.  On average, how often do you smoke cigarettes?  **If response=5, 8 or 9, go to FR87501.**  **If response=1-4, go to BI87345.** | 1 Daily  2 Less than daily, but at least once a week  3 Less than weekly, but at least once a month  4 Less than monthly  5 Not at all  8 Refused  9 Don't know |
| FR87501 | Have you ever smoked cigarettes, even one time? | 1 Yes  2 No  8 Refused  9 Don't know |
| BI87345 | Have you smoked 100 or more cigarettes over your lifetime?  *100 cigarettes= 5 packs of 20 cigarettes OR 4 packs of 25 cigarettes.* | 1 Yes  2 No  8 Refused  9 Don't know |
| FR87216 | On average, how many whole cigarettes do you smoke per day? (1 pack typically contains 20 cigarettes.) | *Enter number of cigarettes.*  88 Refused  99 Don't know |
| FR87226 | On average, how many whole cigarettes do you smoke per week? (1 pack typically contains 20 cigarettes.) | *001 001*  *002 002*  *003 003*  *…*  *100 100*  *101 More than 100*  *888 Refused*  *999 Don't Know*  *Enter number of cigarettes.*  *(Program values of range 1 - 100 with additional text response of 'More than 100'.)* |
| FR87201 | During of the past 30 days, on how many days did you smoke cigarettes? | *Enter number of days. (Range 00-30)*  88 Refused  99 Don't know |
| FR87202 | On average, on days that you smoked cigarettes, about how many cigarettes did you smoke per day? (1 pack typically contains 20 cigarettes.) | *Enter number of cigarettes or choose one of the coded responses.*  88 Refused  99 Don't know |
| FR87118 | How old were you when you first tried cigarette smoking, even one or two puffs? | 88 Refused  99 Don't know |
| FR87510 (Prev. NW87500) | **Ask if (FR87225=2-4) or (FR87501=1).**  At the time when you were smoking cigarettes most often, how often did you smoke them? | 1 Daily or almost daily; most days  2 Less than daily, but at least once a week  3 Less than weekly, but at least once a month  4 Less than monthly  5 Not at all  8 Refused  9 Don't know |
| FR87116 (Prev. NW87501) | **Ask if FR87510=1 or FR87225=1.**  How old were you when you first started smoking cigarettes on most days? | 88 Refused  99 Don't know  *(Range 1 - 87)* |
| FR87131 (Prev. NW87502) | **Ask if FR87510=1.**  During the time when you smoked cigarettes most days, on average, how many cigarettes did you smoke each day? | 001 001  002 002  003 003  …  100 100  101 More than 100  88 Refused  99 Don't know  *Enter number of cigarettes.*  *(Program values of range 1 - 100 with additional text response of 'More than 100'.)* |
| FR87127 (Prev. NW87503) | **Ask if FR87510=1.**  How old were you when you last smoked cigarettes on most days? | 88 Refused  99 Don't know  *(Range 1 - 87)* |
| MK87226 | **Ask all.**  We are now going to ask you about your use of midwakh/dokha.  On average, how often do you smoke midwakh/dokha? | 1 Daily  2 Less than daily, but at least once a week  3 Less than weekly, but at least once a month  4 Less than monthly  5 Not at all  8 Refused  9 Don't know |
| MK87501 | Have you ever smoked midwakh/dokha, even one time? | 1 Yes  2 No  8 Refused  9 Don't know |
| MK87201 | How many times have you smoked midwakh/dokha in your entire life? | 1 Two or fewer  2 3-10 times  3 11-20 times  4 21-50 times  5 51-99 times  6 100 or more  8 Refused  9 Don't know |
| MK87217 | On average, how many times do you smoke midwakh/dokha each day? | 88 Refused  99 Don't know |
| MK87216 | On average, how many times do you smoke midwakh/dokha each week? | 88 Refused  99 Don't know |
| MK87241 | During the past 30 days, on how many days did you smoke midwakh/dokha? | 88 Refused  99 Don't know  *Enter number of days. (Range 00-30)* |
| MK87242 | **Ask if MK87241=1-30.**  On average, on days that you smoked midwakh/dokha, about how many times did you smoke it per day? | 88 Refused  99 Don't know |
| MK87518 | How old were you when you first tried smoking midwakh/dokha, even one or two puffs? | 88 Refused  99 Don't know |
|  |  |  |
| MK87535 (Prev. NW87505) | **Ask if (FR87225=2-4) or (FR87501=1).**  At the time when you were smoking midwakh/dokha most often, how often did you smoke them? | 1 Daily or almost daily; most days  2 Less than daily, but at least once a week  3 Less than weekly, but at least once a month  4 Less than monthly  5 Not at all  8 Refused  9 Don't know |
| MK87519 (Prev. NW87506) | **Ask if FR87510=1 or FR87225=1.**  How old were you when you first started smoking midwakh/dokha on most days? | 88 Refused  99 Don't know  *(Range 1 - 87)* |
| MK87530 (Prev. NW87507) | **Ask if FR87510=1.**  During the time when you smoked midwakh/dokha most days, on average, how many cigarettes did you smoke each day? | 001 001  002 002  003 003  …  100 100  101 More than 100  88 Refused  99 Don't know  *Enter number of cigarettes.*  *(Program values of range 1 - 100 with additional text response of 'More than 100'.)* |
| MK87532 (Prev. NW87508) | **Ask if FR87510=1.**  How old were you when you last smoked midwakh/dokha on most days? | 88 Refused  99 Don't know  *(Range 1 - 87)* |
| HK87209 | **Ask all.**  We are now going to ask you about your use of shisha (also known as waterpipe, hookah, narghile).  Do you smoke shisha…? | 1 Mainly on your own  2 Mainly shared with others  3 Alone and shared, about equally  4 Not at all  8 Refused  9 Don't know |
| HK87226 | **Ask if HK87209=1, 2 or 3.**  On average, how often do you smoke shisha (on your own or in a shared session with others)? | 1 Daily or almost daily; most days  2 Less than daily, but at least once a week  3 Less than weekly, but at least once a month  4 Less than monthly  5 Not at all  8 Refused  9 Don't know |
| NC87802 | Have you ever smoked shisha, even one time? | 1 Yes  2 No  8 Refused  9 Don't know |
| NC87807 | How many times have you smoked shisha in your entire life? | 1 Two or fewer  2 3-10 times  3 11-20 times  4 21-50 times  5 51-99 times  6 100 or more  8 Refused  9 Don't know |
| HK87217 | On average, how many times per day do you smoke shisha? | 88 Refused  99 Don't know  *(Range 1 - 50)* |
| HK87216 | On average, how many times per week do you smoke shisha? | 001 001  002 002  003 003  …  100 100  101 More than 100  888 Refused  999 Don't Know  *(Program values of range 1 - 100 with additional text response of 'More than 100'.)* |
| NC87822 | During the past 30 days, on how many days did you smoke shisha? | 88 Refused  99 Don't know  *Enter number of days. (Range 00-30)* |
| NC87823 | On average, on days that you smoked shisha, about how many times per day did you do that? | 88 Refused  99 Don't know  *(Range 1 - 50)* |
| HK87266 | On average, each time you smoke shisha, how many shisha heads do you smoke? | 88 Refused  99 Don't know  *(Range 1 - 50)* |
| HK87268 | How many shisha heads per week do you usually smoke? | 001 001  002 002  003 003  …  100 100  101 More than 100  888 Refused  999 Don't Know  *(Program values of range 1 - 100 with additional text response of 'More than 100'.)* |
| HK87744 | On average, of those times you smoke shisha, how often does your shisha contain tobacco? | 1 Never  2 Less than half the time  3 Half the time  4 More than half the time, but not always  5 Always  8 Refused  9 Don't know |
| HK87732 | Where do you most often smoke shisha? | 1 At home  2 Coffee shop  3 Bar/ club  4 Restaurant  5 Other, specify  8 Refused  9 Don't know |
| HK87732o | Specify other location: | 88 Refused  99 Don't know |
| NC87810 | How old were you when you first tried smoking ANY shisha (includes any shisha that was smoked on your own or shared), even one or two puffs? | 88 Refused  99 Don't know  *(Range 1 - 87)* |
| HK87535 (Prev. NW87511) | At the time when you were smoking shisha most often, how often did you smoke it? | 1 Daily or almost daily; most days  2 Less than daily, but at least once a week  3 Less than weekly, but at least once a month  4 Less than monthly  5 Not at all  8 Refused  9 Don't know |
| HK87519 (Prev. NW87512) | How old were you when you first started smoking shisha on most days? | 88 Refused  99 Don't know  *(Range 1 - 87)* |
| HK87530 (Prev. NW87513) | During the time when you smoked shisha on most days, on average, how many times a day did you smoke it? | 88 Refused  99 Don't know  *(Range 1 - 50)* |
| HK87532 (Prev. NW87514) | How old were you when you last smoked shisha on most days? | 88 Refused  99 Don't know  *(Range 1 - 87)* |
| ET87091 | **Ask all.**  Thinking about INSIDE YOUR HOME… Over the last 7 days, for how many hours were you exposed to other people’s TOBACCO smoke, including smoke from cigarettes, midwakh, or shisha? | Enter number  88 Refused  99 Don't know |
| ET87092 | **Ask all.**  Thinking about INDOOR PUBLIC PLACES… Over the last 7 days, for how many hours were you exposed to other people’s TOBACCO smoke, including smoke from cigarettes, midwakh, or shisha? (e.g. in school, shops, restaurants, shopping malls, movie theaters.) | Enter number  88 Refused  99 Don't know |
| ET87093 | **Ask all.**  Thinking about OUTDOOR PUBLIC PLACES… Over the last 7 days, for how many hours were you exposed to other people’s TOBACCO smoke, including smoke from cigarettes, midwakh, or shisha? (e.g. at playgrounds, sidewalks, entrances to buildings, parks, beaches.) | Enter number  88 Refused  99 Don't know |
| SL87210 | **Ask all.**  Do you use any tobacco that is not smoked? | 1 Yes, chewing tobacco  2 Yes, Naswar  3 Yes, other  4 No  8 Refused  9 Don't know |

| **Q. No** | **Stem** | **Responses** |
| --- | --- | --- |
| **INTRO 5** | **Now, some questions about you and your family.**  **Press ‘next’ to continue.** | Only one selection  - Next |
| **Y1** | **In which country were you born?** | Select one from   1. United Arab Emirates 2. Other   UN Do not know  DA Prefer not to answer |
| Y2 | **What year did you first come to live in the United Arab Emirates?** | Enter number  OR  UN Do not know  OR  DA Prefer not to answer |
| Y2A | **What was your birth weight (in kg)?** | Weight in kilograms 999 Do not know 888 Prefer not to answer |
| **Y3** | **Were you breastfed when you were a baby?** | Select one from   - 01 Yes - 00 No - UN Do not know - DA Prefer not to answer |
| **Y3A** | **How many months were you breastfed when you were a baby?** | Enter number  OR  UN Do not know  OR  DA Prefer not to answer |
| **Y4** | **When you were 10 years old, compared to average would you describe yourself as:** | Select one from   - 01 Thinner - 02 Plumper - 03 About average - UN Do not know - DA Prefer not to answer |
| **Y5** | **When you were 10 years old, compared to average would you describe yourself as:** | Select one from   - 01 Shorter - 02 Taller - 03 About average - UN Do not know - DA Prefer not to answer |
| **Y6** | **Approximately how much did you weigh (in Kilograms) when you were 18 years old?** | - Weight in kilograms - Do not know - Prefer not to answer |
| **Y7** | **Did your mother smoke regularly around the time when you were born?** | Select one from   - YE Yes - NO No - UN Do not know - DA Prefer not to answer |
| **Y8** | **Did your father smoke regularly around the time when you were born?** | Select one from   - YE Yes - NO No - UN Do not know - DA Prefer not to answer |
| **Y13D** | **Does/did your father ever suffer from? (You can select more than one answer)** | Select from   - HE Heart disease - ST Stroke - BP High blood pressure - DB Diabetes - OB Obesity - CH High cholesterol - NN None of the above - UN Do not know - DA Prefer not to answer |
| **Y16D** | **Has/did your mother ever suffer from? (You can select more than one answer)** | Select from   - HE Heart disease - ST Stroke - BP High blood pressure - DB Diabetes - OB Obesity - CH High cholesterol NN None of the above - UN Do not know - DA Prefer not to answer |
| **Y18** | **How many siblings do you have? (Please include those who have died. Do not include half-sisters, step-sisters or adopted sisters)** | Enter number  OR  UN Do not know  OR  DA Prefer not to answer |
| **Y19** | **Have any of your brothers or sisters suffered from any of the following illnesses? (You can select more than one answer)** | Select from   - HE Heart disease - ST Stroke - BP High blood pressure - DB Diabetes - OB Obesity - CH High cholesterol NN None of the above - UN Do not know - DA Prefer not to answer |
| Y23 | **Before their marriage, what was the family relationship between your father and your mother?** | Select from  00 None  01 First cousin  02 Second cousin  03 Less than second cousin  UN Do not know  DA Prefer not to answer |
| Y24 | **How old were you when you got married?** | - Age - Do not remember - Prefer not to answer |

| **INTRO6** | **Now some questions about your feelings and your mood. Touch next to continue.** | Only one selection  - Next |
| --- | --- | --- |
| **P2A** | **Over the last 2 weeks, how often have you been bothered by any of the following problems?**  **Little interest or pleasure in doing things** | Select from   - 01 Not at all - 02 Several days - 03 More than half the days - 04 Nearly every day - UN Do not know - DA Prefer not to answer |
| **P2B** | **Over the last 2 weeks, how often have you been bothered by any of the following problems?**  **Feeling down, depressed, or hopeless** | Select from   - 01 Not at all - 02 Several days - 03 More than half the days - 04 Nearly every day - UN Do not know - DA Prefer not to answer |
| **P2C** | **Over the last 2 weeks, how often have you been bothered by any of the following problems?**  **Trouble falling or staying asleep, or sleeping too much** | Select from   - 01 Not at all - 02 Several days - 03 More than half the days - 04 Nearly every day - UN Do not know - DA Prefer not to answer |
| **P2D** | **Over the last 2 weeks, how often have you been bothered by any of the following problems?**  **Feeling tired or having little energy** | Select from   - 01 Not at all - 02 Several days - 03 More than half the days - 04 Nearly every day - UN Do not know - DA Prefer not to answer |
| **P2E** | **Over the last 2 weeks, how often have you been bothered by any of the following problems?**  **Poor appetite or overeating** | Select from   - 01 Not at all - 02 Several days - 03 More than half the days - 04 Nearly every day - UN Do not know - DA Prefer not to answer |
| **P2F** | **Over the last 2 weeks, how often have you been bothered by any of the following problems?**  **Feeling bad about yourself, or that you are a failure, or have let yourself or your family down** | Select from   - 01 Not at all - 02 Several days - 03 More than half the days - 04 Nearly every day - UN Do not know - DA Prefer not to answer |
| **P2G** | **Over the last 2 weeks, how often have you been bothered by any of the following problems?**  **Trouble concentrating on things, such as reading the newspaper or watching television** | Select from   - 01 Not at all - 02 Several days - 03 More than half the days - 04 Nearly every day - UN Do not know - DA Prefer not to answer |
| **P2H** | **Over the last 2 weeks, how often have you been bothered by any of the following problems?**  **Moving or speaking so slowly that other people could have noticed? Or the opposite –  being so fidgety or restless that you have been moving around a lot more than usual** | Select from   - 01 Not at all - 02 Several days - 03 More than half the days - 04 Nearly every day - UN Do not know - DA Prefer not to answer |
| **P2I** | **If you have checked off any of these problems in the previous 8 questions, how difficult have these proved for you to do your work, take care of things at home, or get along with other people?** | Select one from:   - 01 Not at all difficult - 02 Somewhat difficult - 03 Very difficult - 04 Extremely difficult - UN Do not know - DA Prefer not to answer |

| **Q. No** | **Stem** | **Responses** |
| --- | --- | --- |
| **INTRO8** | **Now some questions about your health. Please Press ‘next’ to continue.** | Only one selection  - Next |
| **H1** | **In general how would you rate your overall health now ?** | Select one from   - 01 Excellent - 02 Good - 03 Fair - 04 Poor - UN Do not know - DA Prefer not to answer |
| **H1A** | **In general, how was your health in childhood (less than 10 years old)?** | Select one from   - 01 Excellent - 02 Good - 03 Fair - 04 Poor - UN Do not know - DA Prefer not to answer |
| **H2** | **Do you have any of the following?**  **(You can select more than one answer)** | Select from   - 01 Mouth ulcers - 02 Painful gums - 03 Bleeding gums - 04 Loose teeth - 05 Toothache - 06 Dentures - NN None of the above - DA Prefer not to answer |
| **H2A** | **How many permanent teeth (other than wisdom teeth) have you lost?** | Enter number   - UN Do not know - DA Prefer not to answer |
| **H2C** | **Has your dentist told you that you have periodontal disease?** | Select from   - YE Yes - NO No - UN Do not know - DA Prefer not to answer |
| **H2D** | **How often do you use dental floss?** | Select from  - Once daily  - Twice daily  - Three times daily or more  - Two to four times in a week  - Once a week or less  - Never  - Do not remember  - Prefer not to answer |
| **H2E** | **How often do you brush your teeth?** | Select from  - Once daily  - Twice daily  - Three times daily or more  - Two to four times in a week  - Once a week or less  - Never  - Do not remember  - Prefer not to answer |

**WOMEN ONLY QUESTIONS**

| **Q. No** | **Stem** | **Responses** |
| --- | --- | --- |
|  |  |  |
|  |  |  |
|  |  |  |
|  | **Has a doctor ever told you that you have polycystic ovarian syndrome / disease?** | Select from   - YE Yes - NO No - UN Do not know   DA Prefer not to answer |
|  | **How old were you when the doctor first told you that you had polycystic ovarian syndrome / disease?** | Enter number  OR  UN Do not know  OR  DA Prefer not to answer |
|  | **Are you being treated for polycystic ovarian syndrome / disease?** | Select from   - YE Yes - NO No - UN Do not know   DA Prefer not to answer |

**MEN AND WOMEN ONLY QUESTIONS CEASE – ALL PARTICIPANT QUESTIONS – Continued Health**

| **Q. No** | **Stem** | **Responses** |
| --- | --- | --- |
| **L1** | **Has a doctor ever told you that you have had any of the following conditions? (You can select more than one answer)** | Select from   - 04 Obesity - 08 Asthma - 09 Hayfever, allergic rhinitis or eczema - NN None of the above - DA Prefer not to answer |
| L1A | **What was your age when the **** was first diagnosed?**  ***“****” insert each condition from L1 if selected*** | Enter number  OR  UN Do not know  OR  DA Prefer not to answer |
| **L3** | **Has a doctor ever told you that you have diabetes?** | Select one from   - YE Yes - NO No - UN Do not know - DA Prefer not to answer |
| L3A | **Did you only have diabetes during pregnancy?** | Select one from   - YE Yes - NO No - NA Not applicable - UN Do not know - DA Prefer not to answer |
| L3B | **What was your age when the diabetes was first diagnosed?** | Enter number  OR  UN Do not know  OR  DA Prefer not to answer |
| L3C | **Did you start insulin within one year of your diagnosis of diabetes?** | Select one from   - YE Yes - NO No - UN Do not know - DA Prefer not to answer |
| **L5** | **Has a doctor ever told you that you have or had high cholesterol** | Select one from   - YE Yes - NO No - UN Do not know - DA Prefer not to answer |
| **L5A** | **How old were you when the doctor first told you that you had high cholesterol?** | - Age - UN Do not remember - DA Prefer not to answer |
| **L5B** | **Are you being treated for high cholesterol?** | - Diet only - Tablets only - Diet and tablets - No - Prefer not to answer |
| **L6** | **Has a doctor ever told you that you have or had high blood pressure?** | Select one from   - YE Yes - NO No - UN Do not know - DA Prefer not to answer |
| **L6A** | **How old were you when the doctor first told you that you had high blood pressure?** | - Age - UN Do not remember - DA Prefer not to answer |
| **L6B** | **Are you being treated for high blood pressure?** | - 01 Diet only - 02 Tablets only - 03 Diet and tablets - NO No - DA Prefer not to answer |
| **L8** | **Do you regularly take any of the following? (You can select more than one answer)** | Select from:   - 01 Aspirin - 03 Paracetamol - NN None of the above - UN Do not know - DA Prefer not to answer |
| **L9** | **Do you regularly take any PRESCRIPTION medications? (Do not forget medications such as puffers or patches)** | Select one from   - YE Yes – you will be asked about this later by an interviewer - NO No - UN Do not know - DA Prefer not to answer |
| **L10** | **Do you regularly take any of the following? (You can select more than one answer)** | Select from   - 04 Vitamin D - ) - 07 Multivitamins +/- minerals - NN None of the above - DA Prefer not to answer |
| **L10A** | **Do you regularly take any of the following? (You can select more than one answer)** | Select from   - 01 Fish oil (including cod liver oil) - 05 Iron - NN None of the above - DA Prefer not to answer |
| **L11** | **How often have you taken a course of antibiotics in the last year?** | - 01 Never - 02 Once - 03 Twice - 04 Three times - 05 Four times or more - UN Do not remember - DA Prefer not to answer |
| **L11A** | **Were the antibiotics taken in the last month?** | - 01 Yes - 02 No - UN Do not remember - DA Prefer not to answer |
| **L12** | **Have you ever had bariatric surgery for weight loss?** | Select from   - YE Yes - NO No - UN Do not know - DA Prefer not to answer |
| **L12A** | **How old were you when you had the surgery?** | Enter number  OR  UN Do not know  OR  DA Prefer not to answer |
|  | **Has a doctor ever told you that you have Thalassemia?** | Select from   - YE Yes - NO No - UN Do not know   DA Prefer not to answer |
|  | **Has a doctor ever told you that you have Thalassemia trait?** | Select from   - YE Yes - NO No - UN Do not know   DA Prefer not to answer |
|  | **Is this?** | Select from   - α-thalassemia - β-thalassemia - UN Do not know - DA Prefer not to answer |
